# Gut microbiome diversity and high-fibre intake are related to lower long-term weight gain

**DOI:** 10.1038/ijo.2017.66

**Published:** 2017-04-04

**Authors:** C Menni, M A Jackson, T Pallister, C J Steves, T D Spector, A M Valdes

**Affiliations:** 1 Department of Twin Research and Genetic Epidemiology, King's College London, London, UK; 2 Academic Rheumatology, University of Nottingham, Nottingham, UK

## Abstract

**Background:**

Cross-sectional studies suggest that the microbes in the human gut have a role in obesity by influencing the human body’s ability to extract and store calories. The aim of this study was to assess if there is a correlation between change in body weight over time and gut microbiome composition.

**Methods:**

We analysed 16S ribosomal RNA gene sequence data derived from the faecal samples of 1632 healthy females from TwinsUK to investigate the association between gut microbiome measured cross-sectionally and longitudinal weight gain (adjusted for caloric intake and baseline body mass index). Dietary fibre intake was investigated as a possible modifier.

**Results:**

Less than half of the variation in long-term weight change was found to be heritable (h^2^=0.41 (0.31, 0.47)). Gut microbiota diversity was negatively associated with long-term weight gain, whereas it was positively correlated with fibre intake. Nine bacterial operational taxonomic units (OTUs) were significantly associated with weight gain after adjusting for covariates, family relatedness and multiple testing (false discovery rate <0.05). OTUs associated with lower long-term weight gain included those assigned to *Ruminococcaceae* (associated in mice with improved energy metabolism) and *Lachnospiraceae*. A *Bacterioides* species OTU was associated with increased risk of weight gain but this appears to be driven by its correlation with lower levels of diversity.

**Conclusions:**

High gut microbiome diversity, high-fibre intake and OTUs implicated in animal models of improved energy metabolism are all correlated with lower term weight gain in humans independently of calorie intake and other confounders.

## Introduction

Obesity is a growing public health problem that predisposes to cardiovascular diseases and type 2 diabetes. It has been known for many years that obesity has a strong hereditary component and classical twin studies in obesity have reported heritabilities (that is, proportion of inter-individual difference in a trait explicable by genetic variability) on the order of 40–75%.^1^ On the other hand, the biological mechanisms underpinning long-term weight gain or loss, particularly in the context of equal caloric intake, has been less studied. Some studies have indicated a genetic contribution to weight gain^2^ and to metabolic efficiency^3^ over time, but also that non-genetic factors have a significant role in weight gain.

The traditional risk factors for obesity and weight gain are excessive caloric intake,^4^ low physical activity^5^ and low metabolic efficiency.^6^ Animal studies and cross-sectional observational studies in humans have also suggested the role of the composition of the gut microbiome,^7, 8, 9, 10, 11^ in particular lack of microbial diversity.^8^

The term microbiome describes the DNA material of microbial communities within an animal. Humans have around 100 trillion gut microbes that produce a wide range of enzymes, chemicals, hormones and vitamins and potentially interact with their bodies. Under physiological conditions, there is a balance between the intestinal bacteria and the host. Studies have shown that disruption of this intricate system (dysbiosis) and low species diversity are associated with obesity.^7, 12, 13^ Germ-free mice receiving microbiota transplanted from obese donors gained twice as much weight than germ-free mice receiving microbiota from lean donors.^9^ In humans, a recent study from our group found that the presence of one specific bacterial species (*Christensenellaceae*) is associated with lower body mass index (BMI) and that giving this microbe to mice resulted also in lower weight gain.^14^

Research has shown that the largest influence on the gut microbiome comes from diet and the human ability to extract and store calories from food as fat is at least partially impacted by gut microbes.^9^ Gut bacteria generate short chain fatty acids by fermentation of dietary fibre improving insulin sensitivity and fatty acid oxidation.^15^ We hypothesise therefore that microbiome diversity could be influencing the observed relation between dietary fibre and weight gain.

There is, however, little human data on effects of weight change. A greater understanding of alterations of the gut microbiota, in combination with dietary patterns, may provide insights into how the gut microbiota contributes to weight gain and whether it can be exploited as a novel diagnostic, prognostic and therapeutic target in addition to specific microbes, which may be related to BMI. The aim of this study was to assess the association of gut microbiome diversity in adults from the TwinsUK cohort^16^ and change in BMI over time.

## Materials and methods

### Study population

Study subjects were twins enrolled in the TwinsUK registry, a national register of adult twins recruited as volunteers without selecting for any particular disease or traits.^16^ All recruited twins were of the same sex. We analysed data from 1632 females of Caucasian ancestry with BMI assessed on average 9.09 (s.d.= 3.45) years apart, calorie intake (derived from food frequency questionnaires (FFQs)) and physical activity at baseline and microbiome data at follow-up.

The study was approved by NRES Committee London–Westminster, and all twins provided informed written consent.

#### Assessment of weight gain–weight loss

Height and weight were measured using standard scales twice on average 9.09 (s.d.=3.56) years apart. BMI was calculated by dividing weight (in kg) by the square of height (in metres). BMI change per year was calculated adjusting for age, gender, BMI at baseline, calorie intake (derived from FFQs) and physical activity. Physical activity was measured by questionnaire asking their level of activity in a Likert scale (none, light, moderate and intense). Subjects were categorised based on these tertiles The high weight gain group was defined as the top tertile, whereas the low weight gain as the bottom tertile.

#### Fibre and saturated fatty acid intake

Dietary intakes were estimated from a validated 131-item FFQ.^17^ Fibre and saturated fatty acid intakes (g day^–1^) were derived from the UK Nutrient Database,^18^ which provided food content of non-starch polysaccharides (NSP) determined by the Englyst method.^19^ Specifically, fibre and saturated fatty acid intakes were estimated as the consumption frequency of each food multiplied by the nutrient content of the food for the appropriate portion size. Before analysis, fibre and saturated fatty acid intakes were adjusted for the estimated energy intake (kilocalories).^20^

#### Microbiota analysis

A faecal sample was collected at follow-up and the composition of the gut microbiome was determined by 16S ribosomal RNA gene sequencing carried out as previously described.^21^ Briefly, the V4 region of the 16S ribosomal RNA gene was amplified and sequenced on Illumina MiSeq (Illumina Inc., San Diego, CA, USA). Reads were then summarised to operational taxonomic units (OTUs). Quality control was carried out on a per sample basis, discarding paired-ends with an overlap of <200 nt and removing chimeric sequences using *de novo* chimera detection in USEARCH.^22^ *De novo* OTU clustering was then carried across all reads using Sumaclust within QIIME 1.9.0, grouping reads with a 97% identity threshold.^23, 24^ OTU counts were converted to log transformed relative abundances, with zero counts handled by the addition of an arbitrary value (10^−6^). The residuals of the OTU abundances were taken from linear models, accounting for technical covariates including sequencing depth, sequencing run, sequencing technician and sample collection method. These residuals were inverse normalised, as they were not normally distributed, and used in downstream analyses. In order to calculate alpha diversity, the complete OTU count table was rarefied to 10 000 sequences per sample 50 times. Alpha diversity metrics were calculated for each sample in each of the rarefied tables and final diversity measures taken as the mean score across all 50. Alpha diversities were quantified as observed OTU counts and Shannon and Simpson diversity indices. Alpha diversity indexes were standardised to have mean 0 and s.d. 1.

#### Statistical analysis

Heritability of longitudinal weight change was estimated using the software MX^25^ adjusting for age, sex, smoking, calorie intake and physical activities. We estimated heritability using structural equation modelling to separate the observed phenotypic variance into three latent sources of variation: additive genetic variance (A), shared/common environmental variance (C) and non-shared/unique environmental variance (E).^25^ Additive genetic influences are indicated when monozygotic twins are more similar than dizygotic twins. The common environmental component estimates the contribution of family environment, which is assumed to be equal in both monozygotic and dizygotic twin pairs.^26^ The unique environmental component does not contribute to twin similarity, rather it estimates the effects that apply only to each individual and includes measurement error. Any greater similarity between monozygotic twins than dizygotic twins is attributed to greater sharing of genetic influences. Heritability is defined as the proportion of the phenotypic variation attributable to genetic factors, and is given by the Equation, *h*^*2*^=(A)/(A+C+E).

Random intercept logistic regressions were undertaken to evaluate the ability of gut microbial diversity to predict weight gain. Covariates included age, sex, smoking, calorie intake, physical activities, baseline BMI and familiar relatedness. We repeated the analysis adjusting for the above covariates, as well as for use of proton pump inhibitors and antibiotics.

Linear regressions were also undertaken to determine the association between dietary fibre and microbial diversity adjusting for age, BMI, calorie intake, family relatedness and multiple testing.

As we hypothesised that microbiome diversity could be influencing the relation between dietary fibre and weight gain, we repeated the analysis by stratifying the sample between those in the top tertile of Shannon’s diversity (a metric that accounts for abundance and represents species evenness) and those in the bottom tertile.

Logistic regressions were also used to investigate the association between OTU and weight again adjusting for covariates, familiar relatedness and multiple testing using false discovery rate.

Finally, we run partial least square discriminant analysis on OTUs to identify the effects of weight gain and weight loss on the bacterial community using the R package MixOmics. To avoid over-fitting, we evaluated the performance of the model using a 10-fold cross-validation to calculate the area under the curve of the receiver operator characteristics curve.

## Results

The demographic characteristics of the study population are presented in Table 1. Briefly, there were 3718 individuals with longitudinal BMI data available and of those 1662 individuals mainly females with a wide age range (20–74 years at baseline) had microbiome data at follow-up. Heritability analysis^25^ (809 monozygotic pairs and 1050 dizygotic pairs) found that longitudinal weight change has a heritability (*h*^*2*^) of 0.41 (95% confidence interval: 0.31, 0.47), meaning that 59% of the variance in its levels is not defined by a common genetic component.

We then proceeded to investigate the contribution of gut microbiome diversity to this phenotype.

### Alpha diversity

Individuals in the weight gain group had a significantly lower diversity (*P*<0.05) for the Shannon and Simpson indexes, as well as with the observed number of species in spite of having similar BMI at baseline (Table 2). Adjustment for use of proton pump inhibitors and antibiotics did not affect results.

We then investigated one of the dietary factors that has been implicated in microbiome composition, namely dietary fibre intake.^27^

**Figure 1:**
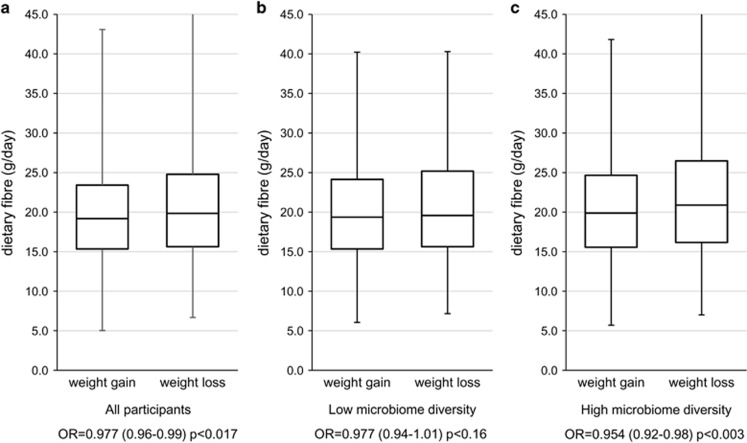
Box plot showing the relationship between dietary fibre intake and weight gain/weight loss (**a**) overall, (**b**) in individuals in the bottom tertile of Shannon’s diversity index and (**c**) in the top tertile of Shannon’s diversity index. The ORs for association with weight gain per gram per day of fibre intake are also shown.

Dietary fibre intake is both positively correlated with measures of microbiome diversity (Shannon: beta (s.e.)= 0.01 (0.004)*, P*=0.002; Table 2) and negatively associated with risk of being in the high weight gain group (odds ratios (OR) (s.e.)=0.977 (0.96–0.99), *P*=0.017; Figure 1a). The association remains even after adjusting for saturated fat intake (OR=0.978 (0.96–0.99), *P*=0.03).

Stratifying the sample between those in the top tertile of Shannon’s diversity and those in the bottom tertile, we found that dietary fibre intake is associated with lower of weight gain among individuals with high gut microbiome diversity (OR=0.954 (0.92–0.98), *P*=0.003; Figure 1c). A similar, although not significant, effect is observed for individuals in the low gut microbiome diversity group (OR=0.977 (0.94–1.01), *P*=0.16). The association between dietary fibre and microbiome diversity remained significant after adjustment for total saturated fat intake (beta=0.012 (0.005), *P*=0.02) and similarly after adjustment for protein intake (beta=0.014 (0.0046), *P*=0.002). We found no association between total protein intake and microbiome diversity (beta=−0.002 (0.002), *P*=0.34).

### OTU abundances that associate with longitudinal weight gain

We identified nine OTUs significantly associated with longitudinal weight gain after adjusting for covariates and multiple testing using false discovery rate <0.05 (Table 3).

Among the bacteria associated with lower risk of weight gain, we found several OTUs from the order *Clostridiales*, in particular of the *Ruminococcaceae* family. As some of these associations may simply reflect a correlation with microbiome diversity, we further adjusted for Shannon’s index. We found that after adjustment for diversity, only six OTUs remained significant, although some only nominally, and that the relative abundance of *Bacteroides* is strongly and negatively correlated with lower microbiome diversity. We also looked for associations at higher taxomic level, and although no significant associations remained after adjusting for multiple testing, the family *Ruminococcaceae* was nominally protective of weight gain (OR=0.89 (0.05), *P*=0.038) in line with the OTU results.

**Figure 2:**
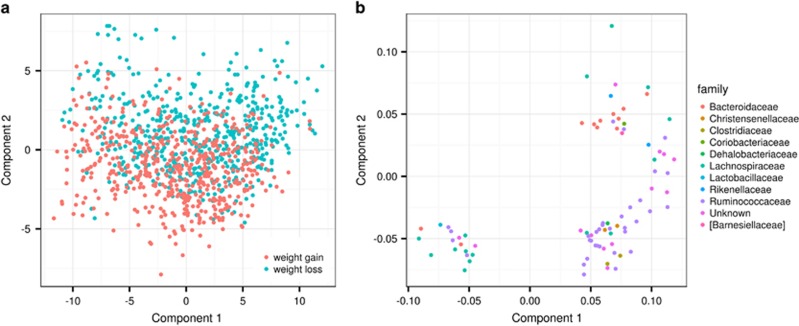
(**a**) Partial least square discriminant analysis score plot based on the relative abundances of OTU in the gut microbiota and their association with weight gain and weight loss. (**b**) Partial least square discriminant analysis loading plot based on the relative abundances of OTU in the gut microbiota and their association with weight gain and weight loss. The OTU with variable influence on projection (VIP) >1 are shown and coloured according to their corresponding family.

Finally, we ran partial least square discriminant analysis to further understand the effects of weight gain and weight loss on the gut bacterial community. The partial least square discriminant analysis analysis showed differences at OTU levels between individuals in the weight gain and weight loss group as depicted in Figure 2, the area under the curve of the receiver operator characteristics curve is 0.57 (s.e.±0.008).

## Discussion

In the largest study to date, we have profiled the effects of gut microbiome diversity and dietary fibre intake on longitudinal weight gain. We showed that long-term weight gain is only in part determined by an individual's genetic make-up and that low gut microbiome diversity is associated with a higher weight gain over time. Our results on longitudinal weight gain are consistent with several studies that have provided evidence of associations between the gut microbiome and cross-sectional measures of body weight.^7, 12, 13^

In this study, the lack of microbiome data at baseline precludes us from being able to assess if higher diversity is a cause or a consequence of higher weight gain. We note two possible interpretations for the data reported. On the one hand, the longitudinal human data presented here is that gut microbiome composition could contribute to weight gain independently of calorie intake, physical activity and other potential confounders (such as, use of proton pump inhibitors or antibiotics).^28, 29^ An alternative interpretation is that weight gain may be contributing to lower bacterial diversity. This second hypothesis requires that at a fixed level of caloric intake, the host metabolism leads to both higher weight gain and lower diversity. However, there is extensive evidence documenting that the microbiome composition influences energy metabolism^30, 31^ and at the same time, to our knowledge there are no proposed mechanisms for slower energy metabolism in the host influencing bacterial composition. If lower bacterial diversity was indeed directly linked to lower weight gain, this would be in agreement with what has been found in murine models regarding the effect of the gut microbiota on energy metabolism in the host^31^ and would suggest that gut microbes may be viewed as ‘novel’ future therapeutic target to treat obesity.

We report that microbiome diversity could be influencing the observed relation between dietary fibre and weight gain. When we stratified the sample between those in the top tertile of Shannon’s diversity and those in the bottom tertile, we found that fibre intake is significantly associated with a decreased risk of being in the high weight gain group among individuals in the high microbiome diversity group but not in those with low microbiome diversity.

We also identified nine OTUs to be significantly associated with weight gain. Adjusting for proton pump inhibitor and antibiotics did not change the results. Conflicting evidence exists regarding phylogenetic signatures in obese human guts, with many studies indicating and increased ratio of *Firmicutes*: *Bacteoridates*,^13, 32, 33, 34^ some showing no trend and some showing the opposite trend.^35, 36, 37^ Here we found that among the eight OTUs that are significantly associated with lower risk of weight gain, seven belong to the *Firmicutes* family, many of them part of the *Ruminococcaceae*. The suggestion that this ratio may not be particularly informative regarding the role of the microbiome in determining body weight had already been put forward by others.^38^

The association between some *Ruminococcaceae* and lower risk of weight gain and *Bacteroides* and higher risk of weight gain may be simply because of their (respectively) positive and negative correlations with microbiome diversity, although in two instances the OTUs remain associated even after adjustment for diversity.

In mice, the gut microbiota is altered during suppression of obesity in a cold environment. *Ruminococcaceae Adlercreutzia* and *Desulfovibrio*^39^ are among the bacteria that increase during this process. Thus, it is possible that *Ruminococcaceae* may be functionally linked to a lean phenotype but further functional studies are needed to assess if this is the case.

A small interventional study in 33 obese individuals identified significant microbiome changes, including a decrease in *Faecalibacterium prausnitzii,* under weight loss in 4 months.^40^ In our data, however, we find no significant association of *Faecalibacterium prausnitzii* with longitudinal weight change, although we find that *Faecalibacterium prausnitzii* correlates cross-sectionally with lower BMI (beta (s.e.)= −0.54 (0.11), *P*=1.4 × 10^−6^) consistent with an association between the abundance of this species in the gut and obesity. We note that we studied a normal population and not an obese group and that this study had a larger study sample and considerably longer follow-up time. However, this suggests that changes in the microbiome in response to weight loss over a short period of time (that is, 4 months) may not reflect differences in microbiome composition associated with lower risk of weight gain over a period of many years.

Not only is weight gain in large part because of non-genetic factors,^1^ but an individual’s gut microbiome diversity is only in part determined by the hosts' genetic make-up. The heritability of gut microbiome diversity has been estimated to range from 0.30 to 0.37,^21^ which means that over 60% of the variation in microbiome diversity is environmentally determined and understanding how to increase microbiome diversity should be a focus of future research.

Our results also suggest that the beneficial effect of fibre on weight may be more pronounced in individuals with higher microbiome diversity, although this may reflect at least in part the fact that individuals, which higher fibre have a greater microbiome diversity.^41^ The healthy effects of a diverse gut microbiome on several phenotypes have already been demonstrated in humans in various settings.^42^ Experimental work in animals has shown that fibre intake reduces the energy density of diet, and the resulting short chain fatty acids promote intestinal gluconeogenesis, incretin formation and subsequently satiety, whereas at the same time short chain fatty acids also deliver energy to the host and support liponeogenesis.^43^ Our data suggest that increasing microbiome diversity may be itself a desirable outcome and that an effect of fibre intake on reduced weight gain is seen more strongly in individuals with higher microbiome diversity.

We note several study limitations, the major one being the lack of measures of microbiome composition at baseline that would enable us to assess the predictive value of diversity with regards to weight gain. Smaller studies, however, have already shown that gut microbiome composition influence weight gain, for example, in children (*n*=25)^44^ and in individuals taking specific antibiotics (*n*=102)^45^ and hence our results are not only consistent but help better document, which OTUs are involved. Another limitation is that the population under study consists of women and there may be gender differences with regards to the role of the microbiome on weight gain. However, this is to our knowledge the largest study to date and the first to explore the association with weight gain over time and not just the association with obesity and leanness. Another limitation of our study is the type of dietary data available from FFQs, which being recall data are subject to some bias. For example, the lack of a significant association between protein intake and microbiome diversity may reflect the limits of FFQ recall data compared with those of carefully controlled dietary intervention studies, hence we cannot exclude the importance of protein intake either on weight gain or on the microbiome from these data.^46^

We also note that the measure of fibre used here referred only to total NSP as the more comprehensive measure was not available. According to the British Nutrition Foundation in the UK, the average intake of NSP is 12.8 g day^–1^ for women and 14.8 g day^–1^ with a recommended average intake for adults is 18 g (NSP) per day.^47^ In our data, the average dietary intake is 20 g NSP per day, which is therefore above the national average and in line with the British National Formulary recommendation.

In conclusion, this study is the first to correlate gut microbiome composition and diversity to long-term (intended as several years) weight change adjusting for calorie intake. It is also one of the largest studies to date linking obesity to the microbiome in humans. Our data are in agreement with other studies that support a role for the gut microbiome composition in the regulation of human body weight, which is to a large extent environmentally determined and independent of caloric intake. As the gut microbiome is modifiable, we believe these results should increase interest in targeting the microbiome for weight control interventions and should encourage research into longitudinal changes in the microbiome in sufficiently powered studies.

## Figures and Tables

**Table 1:** Descriptive characteristics of the study population, overall and by tertiles of weight change

*Variable*	*Overall*	*T1*^a^	*T2*	*T3*
*n*	1632	544	544	544
Women (%)	98.41%	98.90%	98.35%	97.98%
Age at baseline, years	49.76 (8.85)	49.91 (9.49)	50.11 (8.54)	49.25 (8.48)
Age at follow-up, years	58.85 (9.17)	58.03 (9.43)	60.28 (8.79)	58.23 (9.14)
years of follow-up, years	9.09 (3.56)	8·12 (3.83)	10.16 (3·03)	8.98 (3.49)
BMI at baseline, kg m^–^^2^	24.95 (4.17)	25.40 (4.72)	24.02 (3.42)	25.41 (4.13)
BMI at follow-up, kg m^–2^	26.16 (4.58)	24.44 (4.34)	25.24 (3.37)	28.81 (4.69)
BMI change per year, kg m^–2^	0.11 (0.31)	−0.17 (0.26)	0.11 (0.06)	0.39 (0.22)
Fibre intake, g day^–1^	20.4 (6.79)	21.10 (6.98)	20.33 (6.75)	20.02 (6.62)
Kcal intake at baseline	1994.86 (519.32)	2030.46 (526.35)	2015.66 (536.93)	1927.63 (481.66)
Kcal intake at follow-up	1822.56 (528.19)	1858.63 (545.76)	1827.53 (529.07)	1770.76 (500.94)
Protein intake, g day^–1^	80.06 (22.88)	81.02 (24.71)	80.03 (22.54)	79.14 (21.25)
Physical activity low, %	16.63%	19.36%	14.56%	16.28%
Saturated fat intake, g day^–1^	26.05 (10.25)	26.70 (10.49)	26.30 (10.76)	24.98 (9.18)
Smoking (no:ex:yes)	1019:505:108	353:155:36	339:172:33	327:179:39
Use of PPIs	14.15%	13.05%	14.15%	15.26%
				
*Indices of microbiome α-diversity*^b^				
Shannon	5.16 (0.72)	5.21 (0.73)	5.19 (0.73)	5.07 (0.71)
Simpson	0.92 (0.06)	0.93 (0.06)	0.92 (0.06)	0.92 (0.06)
Observed OTU counts	342.12 (97.45)	346.25 (95.70)	348.31 (102.93)	331.79 (93.78)

Mean (s.d.) reported unless indicated otherwise.

Abbreviations: BMI, body mass index; OTU, operational taxonomic unit; PPI, proton pump inhibitor; rRNA, ribosomal RNA.

a T1, T2 and T3 represent, respectively, the first, second and third tertile of change in BMI over time adjusted for age, gender, baseline BMI, calorie intake and physical activity. T3 represents weight gain, whereas T1 represents weight loss.

b The 16S rRNA sequencing data had been summarised to operational taxonomic units (OTUs).^4^ This table was rarefied to a depth of 10 000 OTUs per sample and three measures of gut microbiome alpha diversity were computed: Shannon, Simpson and observed OTU counts.

**Table 2:** Association between indices of microbiome diversity and weight gain, weight loss and dietary fibre intake

	*Weight gain*		*Weight loss*		*Fibre intake (in g)*	
	*OR (s.e.)*	P*-value*	*OR (s.e.)*	P*-value*	*Beta (s.e.)*	P*-value*
Shannon	0.84 (0.05)	9.3 × 10^-4^	1.13 (0.07)	0.03	0.01 (0.004)	0.002
Observed OTU counts	0.85 (0.05)	0.003	1.11 (0.06)	0.1	0.02 (0.01)	0.001
Simpson	0.90 (0.06)	0.05	1.11 (0.07)	0.1	0.01 (0.003)	0.011

Abbreviations: BMI, body mass index; OR, odds ratio; OTU, operational taxonomic unit; PPI, proton pump inhibitor.

Weight gain is defined as the top tertile of the change in BMI over time adjusted for age, gender, baseline BMI, calorie intake and physical activity. Weight loss is the bottom tertile. Analysis are adjusted for PPI and antibiotics use.

**Table 3:** OTUs of the gut microbiome associated with long-term weight gain (ORwtgn) showing the nominal association (*P*) adjusted for age, sex, smoking, calorie intake, physical activity and family relatedness and the FDR *P*-value (Q)

*OTU (taxonomic assignment)*	*OR*_*wtgn*_	*s.e.*	P*-value*	*Q*	*aOR*_*wtgn*_	*as.e.*	*a*P*-value*	*Beta Shannon*	*s.e.*	P-*value*
Firmicutes; c_Clostridia; o_Clostridiales; f_Ruminococcaceae; g_; s_	0.79	0.05	5.8 × 10^−5^	0.03	0.87	0.05	0.018	0.48	0.02	1.9 × 10^−81^
Firmicutes; c_Clostridia; o_Clostridiales; f_; g_; s_	0.81	0.05	1.8 × 10^−4^	0.04	0.86	0.05	0.010	0.43	0.02	1.8 × 10^−75^
Firmicutes; c_Clostridia; o_Clostridiales; f_Ruminococcaceae; g_; s_	0.82	0.04	2.1 × 10^−4^	0.03	0.84	0.05	0.003	0.28	0.02	1.7 × 10^−36^
Firmicutes; c_Clostridia; o_Clostridiales; f_Ruminococcaceae; g_; s_	0.82	0.04	2.4 × 10^−4^	0.03	0.84	0.05	0.001	0.34	0.02	2.5 × 10^−44^
Firmicutes; c_Clostridia; o_Clostridiales; f_Ruminococcaceae; g_; s_	0.81	0.05	2.9 × 10^−4^	0.02	0.91	0.06	0.111	0.50	0.02	5.2 × 10^−85^
Bacteroidetes; c_Bacteroidia; o_Bacteroidales; f_Rikenellaceae; g_; s_	0.82	0.05	3.9 × 10^−4^	0.03	0.91	0.05	0.097	0.33	0.03	1.0 × 10^−32^
Bacteroidetes; c_Bacteroidia; o_Bacteroidales; f_Bacteroidaceae; g_Bacteroides; s_	1.22	0.07	4.3 × 10^−4^	0.03	1.18	0.06	0.002	−0.14	0.02	4.1 × 10^−9^
Firmicutes; c_Clostridia; o_Clostridiales; f_Ruminococcaceae; g_Oscillospira; s_	0.82	0.05	4.6 × 10^−4^	0.02	0.89	0.05	0.032	0.37	0.02	3.6 × 10^−49^
Firmicutes; c_Clostridia; o_Clostridiales; f_Lachnospiraceae; g_Lachnospira; s_	0.82	0.05	4.7 × 10^−4^	0.02	0.89	0.05	0.052	0.37	0.03	2.3 × 10^−43^

Abbreviations: FDR, false discovery rate; OR, odds ratio; OTU, operational taxonomic unit.

The association was then further adjusted for Shannon’s diversity index (aORwtgn). The association between the OTUs relative abundance and Shannon’s diversity index (beta Shannon, s.e. and *P*-value from linear regression).
